# Serum lncRNA-ANRIL and creatinine clearance as cardiovascular risk factors in patients who underwent sleeve gastrectomy

**DOI:** 10.1038/s41598-025-17222-8

**Published:** 2025-09-12

**Authors:** Mohamed Hany, Bart Torensma, Mahmoud Ibrahim, Mohamed N. Roushdy, Anwar Ashraf Abouelnasr, Hebaallah Mohamed Zaki Jaheen, Hala M. Demerdash

**Affiliations:** 1 Department of Surgery, Medical Research Institute, Alexandria University, 165 Horreya Avenue, Hadara, Alexandria, 21561 Egypt; 2 Consultant of Bariatric and Metabolic Surgery, Madina Women’s Hospital, Alexandria, Egypt; 3 Clinical Epidemiology, Erasmus MC, Rotterdam, The Netherlands; 4 Department of Radiology, Alexandria University Student Hospital, Alexandria, Egypt; 5 Alexandria Armed Forces Hospital, Medical Military Academy, Alexandria, Egypt; 6 Blackpool Victoria Teaching Hospital NHS Trust, Blackpool, UK; 7 Department of Clinical Pathology, Alexandria University Hospitals, Alexandria, Egypt

**Keywords:** LncRNA ANRIL, Creatinine clearance, Carotid intima-media thickness, Obesity, Laparoscopic sleeve gastrectomy, Cardiovascular risk, Biochemistry, Genetics, Molecular biology, Physiology, Systems biology, Biomarkers, Cardiology, Endocrinology, Gastroenterology, Medical research, Molecular medicine, Risk factors

## Abstract

Carotid intima-media thickness (CIMT) is a widely recognized marker of subclinical atherosclerosis. Emerging evidence suggests that long non-coding RNAs (lncRNAs), such as ANRIL, may contribute to the development of vascular disease through their roles in inflammation and endothelial dysfunction. We conducted a prospective study involving 93 patients undergoing sleeve gastrectomy. We assessed preoperative and postoperative levels of serum ANRIL and creatinine clearance using the Cockcroft-Gault and Jelliffe formulas. CIMT was measured via duplex ultrasound before surgery and six months postoperatively. We analyzed correlations between changes in ANRIL, renal clearance, and CIMT. Receiver Operating Characteristic (ROC) curves were used to evaluate the diagnostic performance of these markers. Postoperative serum ANRIL levels decreased significantly and were positively associated with reductions in CIMT. Both pre- and postoperative ANRIL levels showed moderate predictive value for CIMT ≥ 1 mm, with an AUC of 0.72 (95% CI 0.61–0.82). Creatinine clearance, calculated by either method, showed lower diagnostic accuracy. This study highlights the potential role of serum lncRNA ANRIL as a biomarker of early vascular improvement following metabolic surgery. Its association with CIMT suggests a link between adipose tissue remodeling and subclinical atherosclerosis regression. However, given CIMT’s moderate predictive value, these findings should be considered exploratory. Further research is needed to determine ANRIL’s clinical utility in cardiometabolic risk stratification.

## Introduction

Obesity is a chronic, multifactorial disease marked by excessive adipose tissue accumulation and strongly linked to cardiometabolic comorbidities, contributing to endothelial activation and a cascade of cellular stress responses^1^. These include heightened oxidative stress^2,3^ and endoplasmic reticulum stress, ultimately impairing endothelial function^4,5^. The ensuing pro-inflammatory state is characterized by elevated circulating cytokines such as IL-1, IL-6, TNF-α, and leptin, alongside chemokines like monocyte chemoattractant protein-1 (MCP-1), which collectively suppress endothelial nitric oxide synthase (eNOS) activity and reduce nitric oxide (NO) bioavailability^6,7^. As NO is a key atheroprotective molecule^8^its depletion fosters vascular dysfunction, intimal thickening, and the development of atheromatous plaques, culminating in progressive atherosclerosis^2,9^. Notably, epidemiologic data underscore an exponential association between increasing BMI and cardiovascular disease (CVD) risk, with each incremental unit above normal BMI conferring approximately a 10% increase in CVD incidence^10–12^.

Long non-coding RNAs (lncRNAs), which are RNA transcripts exceeding 200 nucleotides in length, lack protein-coding potential but engage in complex regulatory interactions by binding specific molecular targets, thereby modulating diverse cellular pathways^13,14^. Mounting evidence supports the involvement of lncRNAs in the pathogenesis of cardiovascular disease^13^.

Among these, the antisense non-coding RNA in the INK4 locus (ANRIL), located on chromosome 9p21.3, exhibits both nuclear and cytoplasmic distribution^15^. This locus has been linked to susceptibility for type 2 diabetes, several malignancies, and atherosclerotic vascular diseases^16,17^. Functionally, ANRIL is implicated in the TNF-α/NF-κB signaling axis, where it is transcriptionally upregulated in response to inflammatory stimuli^18,19^. Elevated ANRIL levels in endothelial cells have been shown to interact with transcription factors such as Yin Yang 1, forming complexes that enhance expression of pro-inflammatory genes, including IL-6 and IL-8 ^18,19^. These mechanisms position ANRIL as a key mediator of endothelial dysfunction and vascular inflammation.

Carotid intima-media thickness (CIMT) is a well-established, non-invasive marker included in the Framingham risk assessment tool for identifying asymptomatic atherosclerosis and quantifying cardiovascular risk^20,21^. In the context of metabolic and bariatric surgery (MBS), including sleeve gastrectomy (SG), CIMT has proven useful in monitoring regression of vascular pathology following weight loss interventions^21^.

In parallel, renal function assessment has emerged as a valuable adjunct in cardiovascular risk stratification^22,23^. Excess adiposity is known to elevate renal metabolic demand, precipitating glomerular hyperfiltration, sodium retention, and susceptibility to kidney injury^24^. While the overlap between cardiovascular and renal dysfunction in obesity, often mediated by dyslipidemia, hypertension, and insulin resistance, is recognized, data remain limited regarding the prognostic utility of creatinine clearance in MBS patients. Nevertheless, oxidative stress-driven inflammation may contribute to renovascular damage through glomerular and vascular hypercellularity^25,26^. As such, creatinine clearance (CrCl), beyond its role in estimating glomerular filtration rate (GFR), may also serve as a surrogate marker for cardiovascular risk in this population.

While ANRIL has been studied in atherosclerosis and type 2 diabetes^27^its dynamic changes in the context of metabolic surgery and its correlation with imaging-based subclinical vascular outcomes remain unexplored. This study addresses this gap by assessing both preoperative and postoperative serum ANRIL expression and its association with CIMT, a validated surrogate of cardiovascular risk.

This study aimed to evaluate the association between serum lncRNA ANRIL and creatinine clearance with CIMT in morbidly obese patients undergoing SG. A secondary aim was to assess the correlation of these biomarkers with anthropometric, inflammatory, and metabolic parameters, and to explore their potential utility as non-invasive predictors of cardiovascular risk before and after surgery.

## Methods

### Study design and participants

This was a single-center, prospective cohort study conducted at the Medical Research Institute, Alexandria University (Egypt), between January 2024 and March 2024, with registration number IORG0008812 E/C. S/N. R11/2023. Eligible participants were adults with obesity scheduled to undergo SG. The study was conducted following the Declaration of Helsinki and received approval from the institutional ethics committee. All participants provided written informed consent before enrollment and data collection.

### Eligibility criteria

Participants were recruited from patients referred to SG during the study period. Inclusion criteria comprised adults aged over 18 years with a body mass index (BMI) > 35 kg/m², or BMI > 30 kg/m^2^ with at least one obesity-associated disease such as type 2 diabetes mellitus, dyslipidemia, or hypertension, following the AMSBS/IFSO 2022 guidelines^28^.

Exclusion criteria included pre-existing chronic kidney disease, established cardiovascular disease, malignancy, coagulopathy, psychological disorders, or active infection.

### Anthropometric assessments

Anthropometric data were collected the day before surgery and at routine follow-ups every three months, including the final visit at 12 months postoperatively. Measurements included weight, height, BMI, waist circumference, hip circumference, and waist-to-hip ratio (WHR), calculated following the World Health Organization standards. Waist circumference cutoffs for increased metabolic risk were > 94 cm for men and > 80 cm for women, while WHR values ≥ 0.90 in men and ≥ 0.85 in women were considered indicative of elevated cardiometabolic risk.

### Surgical technique

All SG procedures were performed by the same surgical team using a standardized approach. Dissection began 6 cm from the pylorus to preserve the gastric antrum, followed by gastric transection over a 40 French bougie, completed with sequential applications of a linear stapler up to the gastroesophageal junction.

### Carotid intima-media thickness measurement

Carotid intima-media thickness (CIMT) was measured bilaterally using high-resolution B-mode ultrasonography (Philips HD 12 Digital Ultrasound System) by experienced radiologists under controlled environmental conditions. CIMT was recorded at three anatomical locations: 1 cm proximal to the carotid bifurcation, 4 cm proximal to the bifurcation (mid-segment), and 1 cm into the bulbous region. The mean of these three measurements was calculated. CIMT values of 1.0 mm or greater were considered indicative of increased cardiovascular risk, as per the American Society of Echocardiography (ASE) guidelines^29^.

### Laboratory investigations

Fasting venous blood samples were collected one week before surgery and at the 12-month postoperative visit. Complete blood counts were analyzed using EDTA-anticoagulated samples. Serum total cholesterol, triglycerides, HDL-cholesterol, LDL-cholesterol, fasting glucose, and creatinine levels were determined using the Hitachi 7180 automatic biochemistry analyzer (Hitachi, Japan). LDL-cholesterol values were calculated using Friedewald’s formula. Serum leptin was measured using a commercial ELISA kit (Cloud-Clone Corp, Cat. No. E-00916hu, USA), while high-sensitivity C-reactive protein (hs-CRP) was assessed via nephelometry (Behring Diagnostics, Germany). Fasting serum insulin was measured using an ELISA kit (DRG International, Cat. No. EIA-2935, USA), and insulin resistance was calculated using the homeostasis model assessment (HOMA-IR), applying the formula: fasting insulin (µIU/ml) × fasting glucose (mmol/L) ÷ 22.5.

### Creatinine clearance estimation

Serum creatinine was measured using an IDMS-traceable Jaffe kinetic assay. Creatinine clearance (CrCl) was estimated using both the Jelliffe and Cockcroft-Gault formulas. The Jelliffe method was calculated as follows:

$${\text{CrCl}}\left( {{\text{ml}}/\min } \right) = \left[ {\left[ {98{-}0.8 \times \left( {{\text{age}}{-}20} \right)} \right] \times } \right[1{-}\left( {0.01 \times {\text{sex}}} \right)\left] { \times \left( {{\text{BSA}}/1.73} \right)} \right] \div \left( {{\text{SCr}} \times 0.0113} \right).$$

The Cockcroft-Gault formula, adjusted for body weight, was calculated as:

$${\text{CrCl}}\left( {{\text{ml}}/\min } \right) = \left[ {\left( {140{-}{\text{age}}} \right) \times {\text{weight}} \times {\text{ }}} \right[1{-}\left( {0.15 \times {\text{sex}}} \right)]] \div \left( {0.814 \times {\text{SCr}}} \right).$$

### Quantification of serum LncRNA ANRIL expression

Peripheral venous blood was centrifuged at 3000 rpm for five minutes, and serum was collected for RNA analysis. Total RNA was extracted using a TRIzol-based method (Invitrogen, Cat. No. 15596-026), and its purity was confirmed using ultraviolet spectrophotometry (UV-1100, PKUCare Industrial Park Technology). RNA integrity was verified by 1% agarose gel electrophoresis. Complementary DNA (cDNA) was synthesized using a commercial reverse transcription kit (Beijing Protein Innovation, Cat. No. BPI01030), and samples were stored at − 80 °C until further use.

Quantitative real-time PCR (qRT-PCR) was performed using a qTOWER3G system (Shanghai Sunshine Biotech). GAPDH was used as the internal reference gene. Primer sequences were as follows: GAPDH forward 5′-GGGAAACTGTGGCGTGAT-3′, reverse 5′-GAGTGGGTGTCGCTGTTGA-3′; lncRNA ANRIL forward 5′-TTATGCTTTGCAGCACACTGG-3′, reverse 5′-GTTCTGCCACAGCTTTGATCT-3′. Amplification conditions included pre-denaturation at 95 °C for 60 s, followed by 40 cycles of 95 °C for 5 s and 60 °C for 15 s. Relative gene expression was quantified using the 2^−ΔΔCT^ method.

### Sample size determination

Sample size was calculated based on expected diagnostic performance for receiver operating characteristic (ROC) analysis. Using the pROC package in R, a minimum of 31 positive and 31 negative cases was required to detect an area under the curve (AUC) of 0.70 with 80% power and an alpha of 0.05. To ensure statistical robustness, a total of 93 participants were recruited.

### Statistical analysis

All statistical analyses were conducted using R software version 4.4.2 and MedCalc version 12.4.0.0. Descriptive statistics included means, standard deviations, and frequencies. Longitudinal changes were assessed using Generalized Estimating Equations (GEE), which accounted for within-subject correlation over time.

Associations between continuous predictors and outcomes (CIMT, ANRIL expression, creatinine clearance) were evaluated using GEE regression models adjusted for time, age, and sex. Logistic regression with GEE was applied to determine the odds of CIMT ≥ 1 mm to biomarker levels. Diagnostic performance was assessed through ROC curve analysis, including AUC, sensitivity, specificity, and optimal cutoff values determined by Youden’s index. Comparisons between AUCs were made to determine the relative predictive power of the studied biomarkers. Statistical significance was defined as a two-tailed p-value < 0.05. Model assumptions, including residual normality and multicollinearity, were assessed using Q-Q plots and variance inflation factors, respectively. GEE was selected due to its ability to account for within-subject correlation over repeated measures and provide population-averaged estimates suitable for translational interpretation.

## Results

### Baseline characteristics

A total of 93 patients were included in the study, with a mean age of 39.6 ± 9.4 years, and the majority were female (82.8%) (Table 1). The mean preoperative body weight was 126.9 ± 23.1 kg, corresponding to a mean BMI of 46.8 ± 7.3 kg/m^2^. The mean waist-hip ratio was 0.95 ± 0.03. Comorbid conditions were common: 62.4% had dyslipidemia, 54.8% exhibited insulin resistance, and 46.2% had hypertension. At baseline, 41.9% of patients had a CIMT of ≥ 1 mm (Table 1).

**Table 1:** Baseline characteristics of the participants (*N* = 93).

Variable	Value
Age	39.6 ± 9.4
Sex	
Female	77 (82.8)
Male	16 (17.2)
Anthropometrics	
Weight (Kg)	126.9 ± 23.1
BMI (kg/m^2^)	46.8 ± 7.3
Waist circumference (cm)	130.2 ± 13.1
Hip circumference (cm)	137.7 ± 11.6
Waist-hip ratio	0.95 ± 0.03
Associated medical illnesses	
Apnea	62 (66.7)
Dyslipidemia	58 (62.4)
Insulin resistance	51 (54.8)
Osteoarthritis	46 (49.5)
Hypertension	43 (46.2)
COPD	24 (25.8)
Menstrual problem	14 (15.1)
GERD	10 (10.8)
Hypothyroidism	9 (9.7)
IBD	6 (6.5)
Congestive Heart failure	4 (4.3)
History of DVT	3 (3.2)
Rheumatoid disease	3 (3.2)
Gout	2 (2.2)
HCV positive	2 (2.2)
HBsAg positive	1 (1.1)
CIMT Sonar (mm)	1.0 ± 0.2
CIMT ≥ 1 mm	39 (41.9)

Cell values represent frequency (%) or mean ± standard deviation. CIMT, carotid intima-media thickness; BMI, body mass index; COPD, chronic obstructive pulmonary disease; GERD, gastroesophageal reflux disease; IBD, inflammatory bowel disease; DVT, deep vein thrombosis.

### Postoperative changes in anthropometry and biomarkers

At one-year follow-up, substantial reductions were observed in anthropometric indices and laboratory markers (Table 2). Mean BMI declined from 46.8 ± 7.3 to 30.5 ± 4.8 kg/m^2^, with a mean weight reduction of 44.0 kg (95% CI −49.6 to − 38.3, *p* < 0.001). Waist and hip circumferences decreased by 30.1 cm and 19.2 cm, respectively, while the waist-hip ratio decreased by 0.10 units (95% CI −0.12 to − 0.09, *p* < 0.001).

**Table 2:** Changes in anthropometrics and lab investigations at post-surgical year 1 estimated by GEE analyses.

Variable	Pre-surgery (*n* = 93)	Year 1 Post-surgery (*n* = 93)	MD (95% CI)	*p*
Anthropometrics				
Weight (Kg)	126.9 ± 23.1	83.0 ± 15.6	-44.0 (-49.6, -38.3)	< 0.001*
BMI (kg/m^2^)	46.8 ± 7.3	30.5 ± 4.8	-16.4 (-18.1, -14.6)	< 0.001*
Waist circumference (cm)	130.2 ± 13.1	100.0 ± 13.0	-30.1 (-33.9, -26.4)	< 0.001*
Hip circumference (cm)	137.7 ± 11.6	118.5 ± 11.6	-19.2 (-22.6, -15.9)	< 0.001*
Waist-hip ratio	0.95 ± 0.0	0.8 ± 0.0	-0.10 (-0.12, -0.09)	< 0.001*
CBC				
Hemoglobin (g/dl)	12.4 ± 1.4	12.5 ± 1.5	0.2 (-0.2, 0.6)	0.412
Platelets (x10^9^/L)	297.5 ± 77.9	253.0 ± 62.6	-44.6 (-64.8, -24.4)	< 0.001*
WBCs (x10^9^/L)	14.6 ± 66.7	6.3 ± 1.9	-8.3 (-21.8, 5.2)	0.228
Lipid profile				
Cholesterol (mg/dl)	216.6 ± 21.9	184.7 ± 11.3	-31.9 (-36.9, -26.9)	< 0.001*
Triglycerides (mg/dl)	191.7 ± 61.6	113.2 ± 20.9	-78.6 (-91.7, -65.4)	< 0.001*
HDL C (mg/dl)	40.9 ± 3.5	51.2 ± 2.9	10.4 (9.4, 11.3)	< 0.001*
LDL C (mg/dl)	138.0 ± 15.9	110.5 ± 7.6	-27.5 (-31.1, -24.0)	< 0.001*
Inflammatory biomarkers				
Leptin (ng/ml)	39.3 ± 6.3	17.3 ± 4.0	-21.9 (-23.5, -20.4)	< 0.001*
hs-CRP (mg/dl)	6.7 ± 2.3	2.5 ± 0.8	-4.2 (-4.7, -3.7)	< 0.001*
Insulin Resistance Assessment				
FBG (mg/dl)	123.0 ± 31.2	89.0 ± 12.3	-34.0 (-40.8, -27.2)	< 0.001*
Insulin (mg/dl)	13.2 ± 8.3	6.2 ± 2.9	-7.0 (-8.8, -5.2)	< 0.001*
HOMA IR	4.2 ± 3.3	1.4 ± 1.3	-2.8 (-3.5, -2.1)	< 0.001*
Creatinine clearance (CrCl)				
Creatinine (mg/dl)	0.8 ± 0.1	0.8 ± 0.1	0.1 (0.0, 0.1)	< 0.001*
CrCl (ml/min) Jelliffe	102.0 ± 16.3	90.2 ± 14.3	-11.9 (-16.2, -7.5)	< 0.001*
CrCl (ml/min) Cockcroft-Gault	140.3 ± 31.1	99.9 ± 22.6	-40.4 (-48.2, -32.6)	< 0.001*
lncRNA ANRIL (fold change)	4.8 ± 2.2	0.8 ± 0.5	-4.0 (-4.4, -3.5)	< 0.001*
pre-CIMT Sonar (mm)	1.0 ± 0.2	0.6 ± 0.2	-0.4 (-0.4, -0.3)	< 0.001*
CIMT ≥ 1 mm (OR)	39 (41.9)	3 (3.2)	0.05 (0.01, 0.16)	< 0.001*

Cell values represent Mean ± standard deviation or frequency (%). ANRIL, non-coding RNA; CIMT, carotid intima-media thickness; WBCs, white blood cells; HOMA IR, homeostatic model assessment of insulin resistance; hs-CRP, high-sensitivity C-reactive protein; FBG, Fasting blood glucose; HDL C, high-density lipoprotein cholesterol; LDL C, high-density lipoprotein cholesterol; BMI, body mass index; CBC, complete blood count; MD, mean difference (Year 1 – Baseline); OR, odds ratio (Year 1 vs. baseline); CI, confidence interval. *Statistically significant (*p* < 0.05).

CIMT improved significantly, with mean thickness decreasing by 0.4 mm (95% CI −0.4 to − 0.3, *p* < 0.001), and the proportion of patients with CIMT ≥ 1 mm dropped from 41.9 to 3.2% (OR: 0.05, 95% CI 0.01 to 0.16, *p* < 0.001).

In terms of metabolic parameters, total cholesterol, triglycerides, and LDL-cholesterol were significantly reduced, while HDL-cholesterol increased by 10.4 mg/dL (95% CI 9.4 to 11.3, *p* < 0.001). Inflammatory markers also improved: leptin levels declined by 21.9 ng/mL and hs-CRP by 4.2 mg/dL (both *p* < 0.001). Insulin resistance improved markedly, with HOMA-IR decreasing from 4.2 to 1.4 (mean difference − 2.8, *p* < 0.001).

Creatinine clearance declined after surgery as expected, with reductions in body weight and muscle mass. By the Cockcroft-Gault formula, CrCl decreased by 40.4 mL/min (95% CI −48.2 to − 32.6, *p* < 0.001); by the Jelliffe method, the reduction was smaller but still significant (− 11.9 mL/min, *p* < 0.001). Serum lncRNA ANRIL expression decreased fourfold (mean difference − 4.0, 95% CI −4.4 to − 3.5, *p* < 0.001).

### Determinants of CIMT and odds of cimt ≥ 1 mm

Multivariable analysis using generalized estimating equations (GEE) revealed several factors associated with changes in CIMT and the odds of having CIMT ≥ 1 mm (Table 3). Higher BMI was independently associated with increased CIMT (MD: 0.01 mm per kg/m^2^, *p* < 0.001) and increased odds of CIMT ≥ 1 mm (adjusted OR: 1.12, 95% CI 1.04 to 1.20, *p* = 0.001). Waist-hip ratio showed a strong positive association with CIMT (MD: 1.08 mm, *p* = 0.003) and an extraordinarily high OR (AOR: 2.7 × 10⁷, 95% CI 10.29 to 7.2 × 10¹³, *p* = 0.023), though this estimate may reflect model instability due to low post-surgical event rates.

**Table 3:** Factors associated with CMIT and odds of having cmit ≥ 1 mm estimated by generalized estimating equations.

Predictor	CMIT (mm)		CMIT ≥ 1 mm	
	MD (95% CI)	p	AOR (95% CI)	p
lncRNA ANRIL (fold change)	0.04 (0.02, 0.06)	< 0.001*	1.36 (1.10, 1.69)	0.004*
Anthropometrics				
BMI (kg/m^2^)	0.01 (0.01, 0.02)	< 0.001*	1.12 (1.04, 1.20)	0.001*
Waist-hip ratio	1.08 (0.37, 1.79)	0.003*	2.7 × 10^7^ (10.29, 7.2 × 10^13^)	0.023*
Creatinine clearance (CrCl)				
CrCl (ml/min) Cockcroft-Gault	0.002 (0.001, 0.003)	< 0.001*	1.03 (1.01, 1.04)	0.001*
CrCl (ml/min) Jelliffe	0.002 (0.000, 0.004)	0.059	1.03 (1.00, 1.06)	0.058
Lipid profile				
Cholesterol (mg/dl)	0.002 (0.001, 0.004)	0.003*	1.02 (1.00, 1.04)	0.034*
LDL C (mg/dl)	0.002 (0.000, 0.004)	0.084	1.02 (0.99, 1.05)	0.145
HDL C (mg/dl)	0.00 (-0.01, 0.01)	0.519	1.00 (0.89, 1.13)	0.959
Triglycerides (mg/dl)	0.001 (0.000, 0.001)	0.073	1.00 (1.00, 1.01)	0.316
Inflammatory biomarkers				
Leptin (ng/ml)	0.01 (0.01, 0.02)	< 0.001*	1.07 (1.00, 1.14)	0.055
hs-CRP (mg/dl)	0.03 (0.01, 0.05)	< 0.001*	1.49 (1.19, 1.87)	0.001*
Insulin resistance				
HOMA-IR	0.02 (0.00, 0.03)	0.007*	1.17 (1.03, 1.33)	0.018*

MD, mean difference in CMIT adjusted for time, age, and sex; AOR, odds ratio of having CMIT ≥ 1 mm adjusted for time, age, and sex. *Statistically significant (*p* < 0.05). CIMT, carotid intima-media thickness; HOMA IR, homeostatic model assessment of insulin resistance; hs-CRP: high-sensitivity C-reactive protein; HDL C, high-density lipoprotein cholesterol; LDL C, high-density lipoprotein cholesterol; BMI, body mass index.

lncRNA ANRIL was significantly associated with CIMT (MD: 0.04 mm per fold increase, *p* < 0.001) and increased odds of CIMT ≥ 1 mm (AOR: 1.36, 95% CI 1.10 to 1.69, *p* = 0.004). Creatinine clearance measured by the Cockcroft-Gault formula was positively associated with CIMT (MD: 0.002 mm per mL/min, *p* < 0.001; AOR: 1.03, *p* = 0.001). The Jelliffe method showed similar trends but did not reach statistical significance (*p* = 0.058).

Among biochemical markers, total cholesterol (*p* = 0.003), leptin (*p* < 0.001), hs-CRP (*p* < 0.001), and HOMA-IR (*p* = 0.007) were all significantly associated with increased CIMT. LDL-cholesterol and triglycerides showed weaker or non-significant associations.

### Predictors of serum LncRNA ANRIL expression

Serum lncRNA ANRIL was significantly associated with BMI and waist-hip ratio (both *p* < 0.01) (Table 4). Higher levels of creatinine clearance, whether estimated by Cockcroft-Gault (*p* < 0.001) or Jelliffe (*p* = 0.014), were positively correlated with ANRIL expression. Elevated cholesterol and LDL-cholesterol were both independently associated with higher ANRIL levels (*p* = 0.002 and *p* = 0.019, respectively).

**Table 4:** Factors associated with LncRNA ANRIL estimated by generalized estimating equations.

Predictor	MD (95% CI)	*p*
Anthropometrics		
BMI (kg/m^2^)	0.16 (0.12, 0.20)	< 0.001*
Waist-hip ratio	7.17 (2.35, 12.00)	0.004*
Creatinine clearance (CrCl)		
CrCl (ml/min) Cockcroft-Gault	0.03 (0.02, 0.05)	< 0.001*
CrCl (ml/min) Jelliffe	0.02 (0.00, 0.04)	0.014*
Lipid profile		
Cholesterol (mg/dl)	0.02 (0.01, 0.03)	0.002*
LDL C (mg/dl)	0.02 (0.00, 0.04)	0.019*
HDL C (mg/dl)	0.03 (-0.05, 0.12)	0.431
Triglycerides (mg/dl)	0.01 (0.00, 0.01)	0.193
Inflammatory biomarkers		
Leptin (ng/ml)	0.12 (0.08, 0.16)	< 0.001*
hs-CRP (mg/dl)	0.43 (0.28, 0.57)	< 0.001*
Insulin resistance		
HOMA-IR	0.27 (0.16, 0.37)	< 0.001*

MD, mean difference in lncRNA ANRIL adjusted for time, age, and sex. CI, confidence interval. *Statistically significant (*p* < 0.05). ANRIL, non-coding RNA; HOMA IR, homeostatic model assessment of insulin resistance; hs-CRP, high-sensitivity C-reactive protein; HDL C, high-density lipoprotein cholesterol; LDL C, high-density lipoprotein cholesterol; BMI, body mass index.

Inflammatory markers leptin and hs-CRP demonstrated strong linear associations with ANRIL (*p* < 0.001), and HOMA-IR was also a significant predictor (*p* < 0.001). These findings support the hypothesis that ANRIL integrates metabolic and inflammatory signaling in the vascular context.

### Determinants of creatinine clearance

BMI and waist-hip ratio significantly influenced CrCl estimated by Cockcroft-Gault (*p* < 0.001 and *p* = 0.014, respectively), whereas the Jelliffe method showed attenuated and mostly non-significant associations (Table 5). Leptin, hs-CRP, and HOMA-IR were significantly associated with CrCl by both methods, though the associations were stronger using the Cockcroft-Gault formula.

**Table 5:** Factors associated with creatinine clearance measured by Cockcroft-Gault and jelliffe formulas estimated by generalized estimating equations.

Predictor	CrCl (Cockcroft-Gault) (ml/min)		CrCl (Jelliffe) (ml/min)	
	MD (95% CI)	*P*	MD (95% CI)	*p*
Anthropometrics				
BMI (kg/m^2^)	2.09 (1.61, 2.58)	< 0.001*	0.32 (-0.04, 0.67)	0.078
Waist-hip ratio	91.92 (18.78, 165.06)	0.014*	32.86 (-44.43, 110.15)	0.405
Lipid profile				
Cholesterol (mg/dl)	0.20 (0.01, 0.38)	0.041*	0.08 (-0.03, 0.20)	0.152
LDL C (mg/dl)	0.23 (-0.03, 0.50)	0.083	0.08 (-0.08, 0.24)	0.325
HDL C (mg/dl)	-0.34 (-1.50, 0.82)	0.567	-0.33 (-0.92, 0.26)	0.269
Triglycerides (mg/dl)	0.05 (-0.06, 0.15)	0.405	0.01 (-0.04, 0.06)	0.783
Inflammatory biomarkers				
Leptin (ng/ml)	1.28 (0.65, 1.90)	< 0.001*	0.32 (-0.09, 0.72)	0.123
hs-CRP (mg/dl)	4.58 (2.67, 6.50)	< 0.001*	1.52 (0.41, 2.63)	0.007*
Insulin resistance				
HOMA-IR	3.25 (1.74, 4.75)	< 0.001*	0.76 (0.02, 1.50)	0.044*

MD, mean difference in CrCl adjusted for time, age, and sex. CI, confidence interval. *Statistically significant (*p* < 0.05). HOMA IR, homeostatic model assessment of insulin resistance; hs-CRP: high-sensitivity C-reactive protein; HDL C, high-density lipoprotein cholesterol; LDL C, high-density lipoprotein cholesterol; BMI, body mass index.

### Diagnostic performance for cimt ≥ 1 mm

**Fig. 1:**
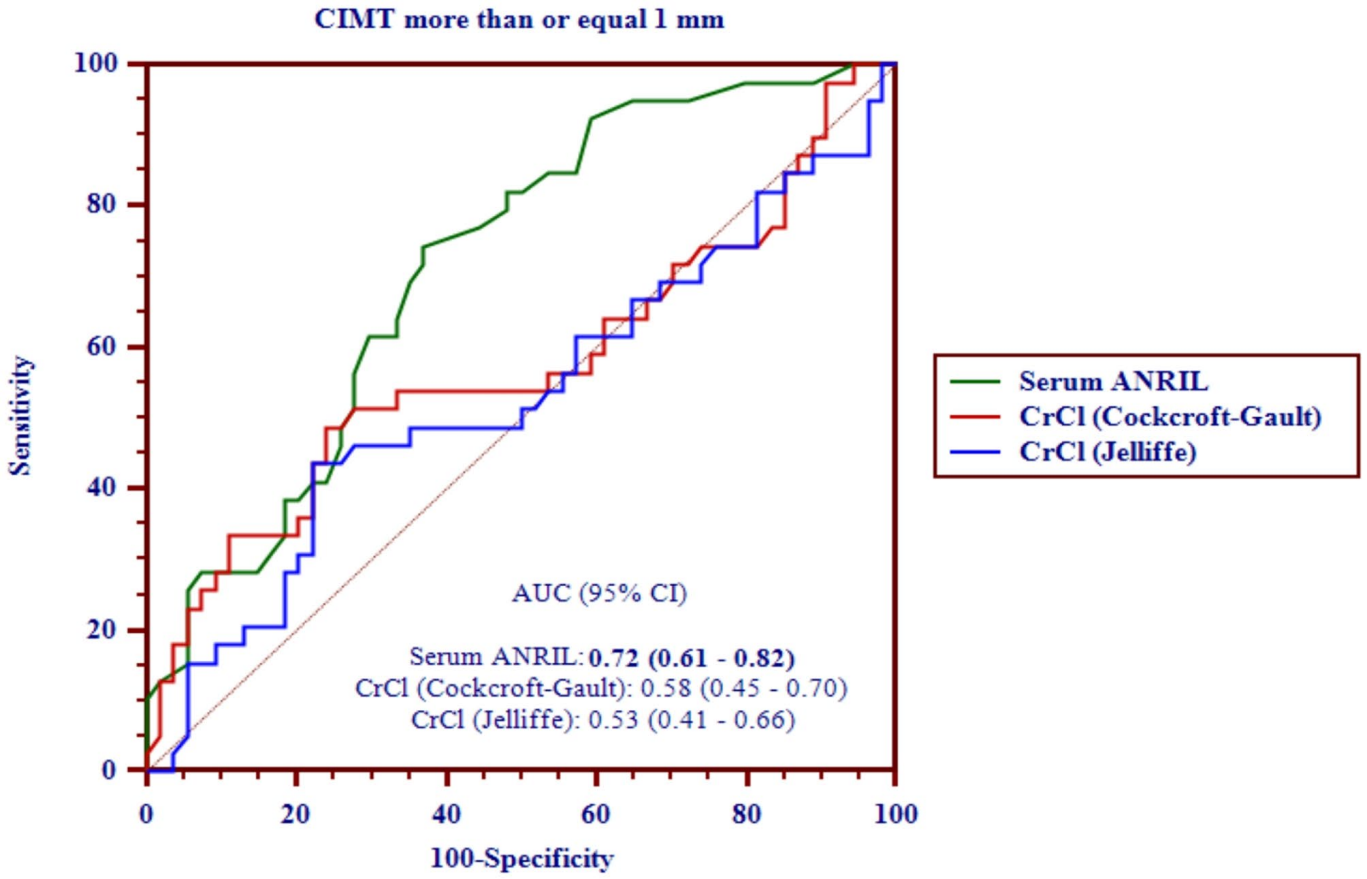
.Receiver operating characteristic (ROC) curves comparing the diagnostic performance of serum lncRNA ANRIL, creatinine clearance estimated by the Cockcroft–Gault formula, and creatinine clearance estimated by the Jelliffe formula in predicting carotid intima-media thickness (CIMT) ≥ 1 mm. The curves illustrate the sensitivity and specificity of each biomarker across a range of thresholds. Serum lncRNA ANRIL demonstrated superior diagnostic accuracy (AUC = 0.72; 95% CI: 0.61–0.82), whereas both creatinine clearance estimates showed lower discriminative performance.

lncRNA ANRIL yielded an AUC of 0.72 (95% CI 0.61 to 0.82), with a sensitivity of 74.4% and specificity of 63.0% at the optimal cutoff (> 3.9-fold change) (Fig. 1) (Table 6). In contrast, creatinine clearance had a limited diagnostic value, with AUCs of 0.58 and 0.53 for the Cockcroft-Gault and Jelliffe methods, respectively (Table 6). The difference in AUC between ANRIL and both CrCl methods was statistically significant (*p* = 0.026 and *p* = 0.023, respectively), confirming the superior discriminative ability of ANRIL (Table 6).

**Table 6:** Diagnostic test accuracy indices of serum LncRNA ANRIL and creatinine clearance measured by Cockcroft-Gault and jelliffe formulas in predicting cimt ≥ 1 mm using the sample lab results at baseline.

CIMT threshold (mm)	Indices	Serum lncRNA ANRIL (fold change)	CrCl (Cockcroft-Gault) (ml/min)	CrCl (Jelliffe) (ml/min)
≥ 1 mm (baseline) Positive cases = 39 Negative cases = 54	AUC	0.72 (0.61–0.82)	0.58 (0.45–0.70)	0.53 (0.41–0.66)
	Best threshold	> 3.9	> 144.1	> 109
	Sensitivity	74.36% (57.9–87.0%)	48.72% (32.4–65.2%)	43.59% (27.8–60.4%)
	Specificity	63.0% (48.7–75.7%)	75.9% (62.4–86.5%)	77.8% (64.4–88.0%)
	PPV	59.2% (44.2–73.0%)	59.4% (40.3–76.6%)	58.6% (38.6–76.8%)
	NPV	77.3% (62.0–88.6%)	67.2% (54.0–78.7%)	65.6% (52.6–77.1%)
	+ LR	2.0 (1.4–3.0)	2.0 (1.1–3.6)	2.0 (1.1–3.6)
	- LR	0.4 (0.2–0.7)	0.7 (0.5–0.9)	0.7 (0.5–1.0)
	ANRIL - Cr Cl CG	∆ AUC (95% CI): 0.14 (0.02–0.26), *p* = 0.026*		
	ANRIL - Cr Cl Jelliffe	∆ AUC (95% CI): 0.18 (0.03–0.34), *p* = 0.023*		
	CrCl CG - CrCl Jelliffe	∆ AUC (95% CI):0.04 (-0.06–0.15), *p* = 0.401		

CIMT, carotid intima-media thickness; AUC, area under the ROC curve; PPV, positive predictive value; NPV, negative predictive value; + LR, positive likelihood ratio; - LR, negative likelihood ratio; CG, Cockcroft-Gault; ∆ AUC, difference in AUC.

## Discussion

Obesity contributes to the pathogenesis of cardiometabolic diseases, notably type 2 diabetes and cardiovascular disease (CVD), both of which show consistent improvement following metabolic surgery^12,18,30^. Nevertheless, the molecular mechanisms linking obesity to vascular dysfunction remain complex, and the identification of robust, non-invasive biomarkers for cardiovascular risk stratification in this population is still evolving^30,31^. In the current cohort, 93 subjects were recruited, and results revealed a female predominance of 82.8%. which was unintentionally designated and could be related to the incidence of increased female patients seeking SG in the present work. However, it is well established that cardiovascular risk is higher in male subjects compared to female subjects due to the presence of the Y chromosome^32,33^. Also, our results revealed obesity-associated comorbidities were highly prevalent, with dyslipidemia in 62.4%, insulin resistance in 54.8%, and hypertension in 46.2% of participants.

Carotid intima-media thickness (CIMT) is an established surrogate for early atherosclerotic change. As a direct ultrasonographic assessment of arterial structure, CIMT correlates with histological alterations and captures cumulative vascular injury, by measuring the thickness of the inner layers of the carotid artery^20,21,34^. Values > 0.7 mm are suggestive of increased vascular risk, while CIMT ≥ 0.9 mm is typically considered pathologic^32^. In our study, SG was associated with a significant reduction in CIMT, with the proportion of patients exhibiting CIMT ≥ 1 mm declining from 41.9 to 3.2% (OR: 0.05, 95% CI 0.01–0.16, *p* < 0.001). Postoperative reductions in BMI, waist-hip ratio, serum cholesterol, and insulin resistance were all positively associated with CIMT improvement, consistent with previous literature^23,35^.

Of particular note, inflammatory markers leptin and hs-CRP showed strong positive associations with CIMT (*p* < 0.001), underscoring the role of chronic low-grade inflammation in obesity-induced endothelial dysfunction. SG appears to attenuate this inflammatory burden, likely contributing to the observed vascular benefit^33,34^.

lncRNA ANRIL has emerged as a potent regulator of cardiovascular pathology, with documented roles in glucose and lipid metabolism, vascular smooth muscle cell behavior, and endothelial cell activation^35,36^. In this study, serum ANRIL levels decreased significantly one year after SG and exhibited a strong correlation with CIMT (MD: 0.04 mm per fold change, *p* < 0.001), supporting its utility as a surrogate marker of subclinical atherosclerosis. Mechanistically, ANRIL modulates VEGF expression and downstream signaling pathways that promote angiogenesis, oxidative stress, and inflammation^40,41^. ANRIL facilitates vascular smooth muscle cell proliferation, enhances cellular adhesion, and impairs apoptosis, collectively fostering plaque formation^36^. It also exerts inflammatory effects through NF-κB activation and cytokine upregulation, particularly IL-6 and IL-8 ^21,36–38^. While CIMT remains a robust structural marker of early atherosclerosis, serum ANRIL offers complementary value by capturing endothelial and inflammatory changes that may precede CIMT-detectable alterations. Furthermore, blood-based measurement of ANRIL is less operator-dependent and more amenable to longitudinal monitoring, enhancing its utility as a screening and follow-up tool^39^.

Consistent with these mechanisms, we found that ANRIL was significantly associated with both BMI (*p* < 0.001) and waist-hip ratio (*p* = 0.004), reinforcing prior evidence linking ANRIL expression with adiposity, particularly under hypercholesterolemic conditions^40^. Epigenetic regulation of ANRIL—such as promoter methylation—has been implicated in early-life programming of adiposity and subsequent metabolic risk^41^. Furthermore, serum ANRIL levels were elevated in individuals with type 2 diabetes and strongly correlated with pro-inflammatory markers leptin and hs-CRP (both *p* < 0.001), in line with findings from studies on diabetic and cancer patients^42–45^. Recent evidence supports this complementary role, showing that combining blood biomarkers with imaging markers yields superior cardiovascular risk assessment compared to either modality alone^39^. ANRIL, as a circulating biomarker, can detect early molecular changes preceding structural vascular alterations, enables repeated monitoring without radiation exposure, and facilitates risk stratification in patients with subtle or subclinical disease. Its blood-based measurement is minimally invasive and feasible for serial assessments. Moreover, ANRIL actively contributes to inflammatory and angiogenic pathways central to atherogenesis, further enhancing its translational utility as a dual biomarker of metabolic and vascular risk^39^. These observations suggest that ANRIL integrates adiposity, inflammation, and vascular dysfunction, and may represent a therapeutic target in metabolic disease^46^.

Serum cholesterol and LDL-cholesterol were both significantly associated with ANRIL levels (*p* = 0.002 and *p* = 0.019, respectively), mirroring studies that describe a link between ANRIL expression and hypercholesterolemia^47,48^. In contrast, ANRIL was not significantly associated with HDL-cholesterol or triglyceride levels.

Regarding renal parameters, creatinine clearance (CrCl) was calculated using both the Cockcroft-Gault and Jelliffe formulas. The Cockcroft-Gault formula, after adjustment for body weight, considers factors such as weight, age, and sex. While the Jelliffe method considers body surface area (BSA). Results revealed a significant postoperative decline in CrCl by both formulas, consistent with evidence from prospective studies showing that MBS reverses obesity-associated hyperfiltration and improves renal hemodynamics through reductions in glomerular perfusion and systemic vascular resistance^49^.

Glomerular hyperfiltration in obesity represents a maladaptive hemodynamic response involving increased renal blood flow, elevated filtration pressure, sodium retention, activation of the renin-angiotensin-aldosterone system, and adipose tissue-derived inflammatory and hormonal signals, such as leptin and TNF-α, which promote endothelial dysfunction and renal injury^49^. This could be explained by obesity-induced glomerular hyperfiltration prompted through a constellation of mechanisms, including altered glomerular hemodynamics, upregulated adipokines, and oxidative stress^49–51^. It is worth mentioning that other factors that were not addressed in either formulas could be responsible for causing the observed decline in CrCl after weight loss as estimated loss of muscle mass, as well as dietary and lifestyle modifications. However, in this study, it was assumed that excess body weight in obesity is mostly adipose tissue, which does not produce creatinine. According to a proportional relationship, an obese subject losing 10% of body weight would display a 10% reduction of the GFR^52,53^. Furthermore, only the Cockcroft-Gault estimate showed a significant association with CIMT ( *p* < 0.001), suggesting its potential superiority in reflecting renal–vascular interplay. This may be attributed to its incorporation of actual body weight, making it more sensitive to metabolic load and changes in body composition after bariatric surgery, as previously suggested by Basolo et al.^49^.

In this context, glomerular hyperfiltration is considered a sign of intraglomerular hypertension that might induce albuminuria and may advance to glomerulosclerosis^54^. Also, leptin levels, which were strongly linked to CrCl in our study (*p* < 0.001), may further exacerbate glomerular injury by inducing vascular inflammation, hypertrophy, and fibrosis via TGF-β1 pathways^55–57^.

Importantly, hs-CRP and HOMA-IR also demonstrated strong associations with creatinine clearance, highlighting the convergence of inflammation, insulin resistance, and renal hyperfiltration in obesity-related cardiovascular risk^56–59^.

A noteworthy finding of this study is the significant correlation between serum ANRIL and creatinine clearance as estimated by both Jelliffe (*p* = 0.014) and Cockcroft-Gault (*p* < 0.001) methods. These findings support prior data linking oxidative stress and inflammatory signaling to both endothelial and renal dysfunction^52^. In particular, ANRIL expressions in plasma and atherosclerotic plaques have been shown to reflect disease severity in coronary artery disease^53^.

The ROC curve analysis reinforced the clinical utility of ANRIL as a diagnostic biomarker for elevated CIMT (AUC = 0.72, sensitivity 74.4%, specificity 63%), outperforming CrCl, which demonstrated poor discriminative capacity regardless of the formula used. Notably, the diagnostic threshold for ANRIL (> 3.9-fold change) was derived from baseline measurements using CIMT ≥ 1 mm as the endpoint. It’s acceptable AUC and balance between sensitivity and specificity support its utility in distinguishing individuals at higher preoperative vascular risk, independent of postoperative improvement.

Although serum ANRIL levels were associated with increased CIMT and showed acceptable discriminatory performance for identifying CIMT ≥ 1 mm, the clinical applicability of ANRIL as a predictor of cardiovascular events remains limited by the moderate predictive capacity of CIMT itself. These findings should thus be interpreted as exploratory and hypothesis-generating, warranting further investigation using larger cohorts and mediation modeling approaches.

The strengths of this study include the prospective design, standardized measurement of CIMT, and laboratory markers. To our knowledge, this is among the few studies to evaluate lncRNA ANRIL in weight loss and vascular remodeling following MBS. However, limitations must be acknowledged; firstly, 82.8% of the studied subjects were females, which may influence biomarker interpretation. This emphasizes the need to perform the study on a larger, multiethnic cohort. Secondly, further studies are required to cautiously evaluate CrCl by other methods that evaluate muscle mass loss, as well as dietary and lifestyle modifications that may be implicated in decreased CrCl after SG. Additionally, existing literature on the triad of CIMT, glomerular hyperfiltration, and subclinical atherosclerosis in the MBS population remains limited, warranting further large-scale investigations.

While our findings suggest that serum lncRNA-ANRIL and creatinine clearance may hold promise as markers of cardiovascular risk after sleeve gastrectomy, their clinical utility remains to be established. Larger prospective studies with longer follow-up are warranted to validate these associations and to determine whether ANRIL can provide incremental prognostic value beyond established risk scores. Integrating molecular biomarkers with functional and imaging parameters may ultimately help refine individualized cardiovascular risk stratification in bariatric populations.

## Conclusion

This study highlights serum lncRNA ANRIL as a potential biomarker for vascular improvement following MBS. Its association with reductions in CIMT suggests a mechanistic link to subclinical atherosclerosis regression. Considering the potential use of serum lncRNA ANRIL as a sensitive and non-invasive biomarker, it is especially relevant in conditions where expected changes in CIMT may be subtle, particularly when measured in plaque-free areas. Additionally, evaluating lncRNA ANRIL requires only a simple blood sample, which facilitates ongoing monitoring over time, particularly for individuals identified as having an increased risk at an early stage.

However, given the modest sample size and lack of external validation, the clinical utility of ANRIL should be interpreted cautiously. Larger studies are needed to confirm these findings and determine whether ANRIL can reliably complement existing cardiovascular risk models or inform postoperative monitoring strategies.

Figure 1: Receiver operating characteristic (ROC) curves comparing the diagnostic performance of serum lncRNA ANRIL, creatinine clearance estimated by the Cockcroft–Gault formula, and creatinine clearance estimated by the Jelliffe formula in predicting carotid intima-media thickness (CIMT) ≥ 1 mm. The curves illustrate the sensitivity and specificity of each biomarker across a range of thresholds. Serum lncRNA ANRIL demonstrated superior diagnostic accuracy (AUC = 0.72; 95% CI 0.61–0.82), whereas both creatinine clearance estimates showed lower discriminative performance.

## Data Availability

The datasets generated and analyzed during the current study are available from the corresponding author on reasonable request.
